# Risk Reduction of Intrahepatic Abscess After Locoregional Therapy for Liver Cancer in Patients with Prior Hepatobiliary Intervention

**DOI:** 10.3390/diagnostics15030333

**Published:** 2025-01-30

**Authors:** Peter Rudnick, Kaleb Feia, Paul Laeseke, Joseph Herman, Jeff Geschwind

**Affiliations:** 1 School of Medicine, Creighton University, Omaha, NE 68178, USA; prr43486@creighton.edu; 2 Department of Biochemistry and Molecular Biology, University of Iowa, Iowa City, IA 52242, USA; kaleb-feia@uiowa.edu; 3 Department of Radiology, University of Wisconsin School of Medicine and Public Health, Madison, WI 53792, USA; plaeseke@wisc.edu; 4 Department of Radiation Oncology, Northwell Health, New York, NY 11042, USA; jherman1@northwell.edu; 5 Division of Oncology, USA Oncology Centers, Northbrook, IL 60062, USA

**Keywords:** hepatic abscess, bilioenteric anastomosis, biliary stent, antibiotic prophylaxis, bowel preparation, intraarterial therapy, local ablative therapy

## Abstract

Intrahepatic abscess is an exceedingly rare complication of locoregional therapy for patients with liver cancer. However, in patients who underwent prior hepatobiliary intervention, the incidence of liver abscess increases significantly, causing morbidity and even mortality in such patients. Here, we will review the relative risk of developing a liver abscess after intraarterial and ablative locoregional therapies in patients with liver cancer depending on whether they underwent any kind of prior hepatobiliary procedures that resulted in violation of the Ampulla of Vater. As a result, patients deemed at high risk of developing a liver abscess were treated prophylactically, with the combination of bowel preparation and antibiotics nearly eliminating the occurrence of a liver abscess after locoregional therapy. Therefore, given the significant risk of developing a liver abscess in patients with prior hepatobiliary procedures, management consisting of prophylactic bowel preparation with antibiotic coverage followed by antibiotics post-locoregional therapy is recommended.

## 1. Introduction

Locoregional therapies for patients with primary and secondary liver cancer are generally safe and effective. Although these therapies may not be appropriate for all stages of liver cancer, they all share a remarkably high safety profile, especially regarding the risk of developing a liver abscess post-procedure. However, in patients who have undergone prior hepatobiliary procedures that compromise the Ampulla of Vater, such a risk increases significantly, resulting in worsening patient morbidity and mortality [1]. Stenting across the ampulla and bilioenteric anastomosis are two common examples of biliary interventions associated with the development of liver abscesses after locoregional therapies because they disrupt the sphincter of Oddi, which prevents the backflow of bacteria from the small bowel into the liver [1]. When the sphincter is no longer intact, bacteria flow freely into the intrahepatic biliary tree, which itself is extremely susceptible to an ischemic insult, as the biliary tree is predominantly supplied by the hepatic artery. Ischemia-inducing locoregional therapies increase the likelihood of abscess formation as bacteria invade necrotic tumor tissue via the biliary tree, where they can proliferate into an abscess.

Given the significantly higher likelihood of developing an abscess under these conditions, patients who underwent any hepatobiliary procedure should be treated prophylactically with a combination of bowel preparation and antibiotics. Final recommendation about the ideal and most effective therapeutic regimen to prevent the formation of a liver abscess rests with the infectious disease department and is largely institution-dependent, as no consensus exists about a standard protocol.

## 2. Risk of Liver Abscess in Patients Without Prior Hepatobiliary Intervention

The occurrence of liver abscesses in patients who have not previously undergone any reconstructive hepatobiliary procedures is an uncommon complication of locoregional therapies. This can be attributed to the intact nature of the sphincter of Oddi [1], which prevents any colonization of the intrahepatic biliary tree from bacteria in the duodenum. The incidence of a liver abscess for such patients ranges from 0.2% to around 6% depending on the locoregional procedure that was performed (Table 1).

For patients who underwent conventional transarterial chemoembolization (TACE) procedures, the rate of liver abscess varied between 0.2% [2] and 0.7% [2,3,4]. One study, clearly an outlier, that used drug-eluting beads as opposed to lipiodol for the TACE regimen reported a rate of 8.8%. Such a high rate was attributed to the more profound ischemia and degree of inflammation caused by the chemotherapy-carrying beads than that by conventional TACE with lipiodol. Given the extreme sensitivity of the biliary tree to any ischemic insult because of its reliance on the arterial supply, some have suggested that technical considerations such as angiographic endpoints should be used when TACE with drug-eluting beads is performed.

Similarly, the rate of liver abscess was very low for patients who underwent radioembolotherapy with yttrium-90 (1/15 patients or 6.6%) [5].

The incidence of liver abscess after percutaneous ablative therapy is even lower than after intraarterial therapies, ranging from 0.3 to 1.8% [6,7,8,9,10]. Aggressive antibiotic therapy both prophylactically and after the procedure prevented hepatic abscess in all 176 patients, confirming the role of antibiotic therapy in eliminating the risk of developing a liver abscess in such patients [11].

Microwave ablation (MWA) has shown an excellent therapeutic efficacy and safety profile in patients with primary and metastatic liver tumors [1]. The rate of liver abscess was reported to be less than 0.4% [12,13]. The rate of liver abscess was similarly low at 0.7% when patients were treated with laser-induced thermotherapy (LITT) [14]. Although limited, data on radiation therapy revealed a similarly low rate of hepatic abscess (one abscess in 28 patients treated with liver radiation) [15], and occasional case reports of cases of liver abscess after stereotactic body radiation therapy [16]. A systematic review of surgical resection for colorectal liver metastases reported an abscess rate of 3% [17].

Multiple studies that were designed to compare various therapeutic modalities such as RFA vs MWA or TACE vs RFA also reported abscess rates between 0.2 and less than 5% [18,19,20,21]. Table 1 outlines the incidence of liver abscess rates across treatment modalities.

## 3. Risk of Liver Abscess in Patients with Prior Hepatobiliary Intervention

The risk of developing an intrahepatic abscess is extremely low after any locoregional therapy for liver cancer (intraarterial or percutaneous ablative therapies), especially in the absence of any prior reconstructive hepatobiliary surgery or biliary interventions such as biliary stenting. As long as the Sphincter of Oddi is preserved, the risk is negligible. This is not the case, however, when patients have undergone procedures that result in the compromise of the Ampulla of Vater, such as biliary stents either placed endoscopically or percutaneously, as well as surgical procedures including Whipple or reconstructive hepatobiliary reconstructive surgery (hepaticojejunostomy or choledochojejunostomy, for example). The incidence of liver abscess in such patients increased significantly to between 10.6% and 100% (see Table 2). When they occurred, most abscesses were diagnosed within 21 days of a locoregional procedure.

In such a patient population, the incidence of liver abscess after TACE increased dramatically to 12–86% depending on the type of surgical procedures performed (Whipple procedure, pylorus-preserving pancreaticoduodenectomy (PPPD), PTBD, or choledochojejunostomy) [2,3,4,5,22]. Antibiotic prophylaxis did not significantly reduce abscess rates in these patients [23]. Additionally, patients with metastatic liver tumors were significantly more likely to develop liver abscesses than patients with hepatocellular carcinoma [4].

The rate of liver abscess in patients with a compromised biliary tree with yttrium-90 radioembolization was lower than that seen with TACE (7%), likely due to the less profound degree of ischemia than what is encountered with TACE. This difference was even more evident in a comparative study between TACE and Y90 radioembolization, where the rate of liver abscess was 23% in the TACE arm and 0% in the Y90 cohort, highlighting the key technical differences in terms of embolization and resultant ischemia between the two therapies [24,25].

As with TACE, treatment with percutaneous thermal ablation in the form of RFA or MWA resulted in high rates of liver abscesses ranging from 22% to 100% of patients, including some who were pre-treated with the antibiotics listed in Table 3 [6,7,13,26,27,28,29].

Remarkably, bowel preparation and aggressive prophylactic antibiotic therapy significantly reduced the liver abscess rate in this highly vulnerable patient population from 60% to 0%, suggesting that bowel preparation combined with antibiotic therapy administered prophylactically is an effective approach to reduce, if not eliminate, the risk of developing a liver abscess [1].

The risk of developing a liver abscess after locoregional therapy is equally high at around 22% in patients who had previously undergone a Whipple procedure [15]. The risk was highest with TACE (34.8%) and lowest with ablative treatment (8.7%) [15].

**Table 3:** Sample antibiotic regimens across locoregional therapies.

Treatment Modality	Timing: Antibiotics (Dosing)	Bowel Preparation	Abscess Rate
Radiofrequency Ablation [6]	D0: Ceftriaxone (2 g) + Metronidazole (1 g) D0 to D4: Piperacillin-tazobactam (4 g × 2)	No	4/13 (31%)
Microwave Ablation (Group A) [1]	D0: Cefazedone (2 g × 4) + Amoxicillin-flucloxacillin sodium (2 g × 2) + Levofloxacin (0.5 g)	No	6/10 (60%)
Microwave Ablation (Group B) [1]	D(−1): Metronidazole (0.4 g × 3) + Gentamicin sulphate (8 WU × 3) D0 to D4: Imipenem cilastatin sodium (1 g × 2) + Linezolid (0.6 g × 2)	Yes	0/10 (0%)
Transarterial Chemoembolization [30]	D(−1) to D1: Tazobactam/piperacillin (3.375 g × 4)	Yes	0/4 (0%)
Yttrium-90 Radioembolization [24]	D(−2) to D13: Levofloxacin + Metronidazole	Yes	0/16 (0%)

Regardless of the type of hepatobiliary procedures, patients who undergo any locoregional therapy in such a context are at a significantly higher risk of developing a liver abscess, making this group of patients extremely vulnerable. This is why such patients should be carefully evaluated while weighing the benefit/risk ratio of locoregional therapy before performing such locoregional therapy.

## 4. Prophylactic Regimens

Given the significantly higher risk of developing a liver abscess when a locoregional treatment is performed on a patient who has undergone a prior hepatobiliary procedure, it is imperative to consider pre-treating such patients with a combination of bowel preparation and antibiotics. Fortunately, data about the success of such a prophylactic combination treatment exist. Here, the selection of antibiotics can make a significant difference, as reported in a study of patients treated with TACE. When patients received prophylactic cephalexin, all developed an abscess (4/4 patients), whereas the combination of piperacillin and tazobactam in addition to bowel preparation prevented the occurrence of any liver abscess in 0 out of 4 patients [30]. Neither antibiotics nor bowel preparation caused significant side effects that necessitated discontinuing the regimen, or would prevent their use as a prophylactic regimen. In most clinical trials, metronidazole was the antibiotic agent of choice because of its coverage against amoebic organisms such as Entamoeba histolytica. The combination of a third or fourth generation cephalosporin, aminoglycoside, and metronidazole was a regimen that could be prescribed for up to 6 weeks after locoregional therapy if such an antibiotic was needed. Overall, various antibiotic regimens were preferred across several clinical trials—summarized in Table 3—where a particular regimen may have been selected because of patient allergies or simply the unavailability of a specific drug. Yet, despite such issues, the key to selecting a successful antibiotic treatment resides in its ability to cover the main types of organisms that reside in the bowel and upper intestinal tract.

## 5. Diagnosis and Management of Liver Abscesses

As discussed above, high-risk patients treated with locoregional therapy for liver cancer should be watched closely in the periprocedural period for the development of liver abscess. Clinical signs are typically nonspecific, including abdominal pain especially in the right upper quadrant, fever, weight loss, jaundice, and inflammation [18]. In some cases, especially when extensive and associated with biliary etiologies, liver function tests can be abnormal. As a result, given the lack of specific symptoms, a diagnosis is generally established using medical imaging [18]. However, in patients at high risk of developing a liver abscess post-TACE or RFA, a diagnosis is often first made based on clinical and laboratory findings, especially given the high index of suspicion before being confirmed with imaging [18].

Ultrasound is the imaging modality of choice to diagnose liver abscesses and possibly identify the etiology, as it combines high diagnostic accuracy with ready availability. In addition, it offers the added advantage of being noninvasive and relatively inexpensive, and does not require either contrast media or ionizing radiation [31]. Liver abscesses generally appear as poorly delineated with variable echogenicity, from hypoechoic containing internal echoes to hyperechoic, and without any evidence of perfusion centrally on color Doppler imaging consistent with liquefactive necrotic tissue centrally. When gas bubbles are present, the diagnosis is easily established.

Despite the clear benefits of ultrasound, contrast-enhanced CT imaging, through its greater sensitivity that that of ultrasound (>90%), remains the absolute gold-standard in terms of imaging. However, given the need for contrast media and exposure to radiation, CT is reserved for more complex situations such as post-operative patients, or after locoregional therapies for liver cancer patients [31]. Although liver abscess can have a varied appearance on CT, features most commonly present include peripheral enhancement surrounding a fluid-filled central area of hypoattenuation, which can create an almost pathognomonic “ring” or “double target sign”. As with ultrasound, the presence of gas bubbles within the abscess cavity would confirm the diagnosis. It is also interesting to note that the appearance on CT matches the phase of the abscess; from pre-suppurative to suppurative, with imaging features evolving from heterogenous, hypoattenuating, ill-defined tumor-like mass or masses to well delineated, thick rim-enhancing, hypodense central areas that no longer mimics tumors.

Other imaging techniques such as magnetic resonance imaging (MRI) are rarely used to establish a diagnosis of liver abscess. Only when a biliary cause for the abscess is suspected can an MRI be helpful in identifying the level of biliary obstruction responsible for the formation of the abscess. Colonoscopy can also be used if a gastrointestinal septic source is suspected [18].

Immediately after a TACE or RFA procedure, imaging can show air within an area of necrosis from the treatment. An increased quantity of air, or air at a site distant from the treatment zone leads to suspicion of liver abscess [18]. On CT, abscesses were described as low-attenuation lesions with thick walls, within embolized tumors [4].

Liver abscesses are typically classified based on the type of pathogens responsible for the infection, such as bacterial, parasitic, mixed, or fungal [18]. In Western countries, the vast majority of liver abscess are caused by bacteria. When a liver abscess occurs as the complication of locoregional therapy, Gram-negative or Gram-positive bacteria of intestinal sources are most commonly the culprit pathogens for the abscess [1]. More specifically, after a TACE procedure, *Escherichia coli* (52.4% of patients), *Enterobacter cloacae* (38.1%), and *Enterococcus faecalis* (28.6%) were the most common pathogens. Furthermore, a third of these patients developed polymicrobial purulent cultures [4]. Timely identification of the responsible microorganism is crucial for prompt initiation of the appropriate targeted antibiotic therapy to improve patient outcomes. Whereas obtaining positive cultures from the purulent abscess collection in all patients has been reported, the same could not be said about blood cultures. Indeed, only approximately half (47.6%) of blood cultures yield positive results [4], thereby highlighting the importance of collecting a sample of the purulent material directly from the pyogenic liver abscess, as relying on blood culture alone could fail to identify the responsible organism.

Liver abscesses can also be classified by their anatomic origin or route of infection, such as biliary (direct extension from surrounding infected tissues), portal venous circulation, arterial hematogenous-causing systemic bacteremia, or traumatic [18,31]. As already explained above, although locoregional therapies such as TACE and ablation can theoretically cause liver abscesses through induction of tumor necrosis in the nidus of the tumor, the incidence is low, less than 5%, unless prior manipulation was performed at the junction between the hepatic biliary system and the small bowel. Such cases of liver abscess are biliary in origin, as they are the direct result of an ascending infection from the bowel to the intrahepatic biliary tree through a compromised and/or damaged sphincter of Oddi. When a liver abscess occurs after TACE or an ablation, the diagnosis is usually established clinically. The index of suspicion should only be high if the patient was at high risk of developing an abscess in the first place. Patients with neuroendocrine tumors are especially vulnerable to an ascending infection through the biliary tree, given the healthy nature of the liver parenchyma, unlike patients with cirrhosis and HCC.

Standard management of liver abscess consists of systemic antibiotic therapy that can be combined with image-guided percutaneous drainage [31]. If the abscess is small, generally less than 3 cm, uniloculated systemic antibiotic therapy alone may be sufficient to fully treat the abscess, especially if it was started promptly [9]. Signs of post-treatment success include pain relief, normalization of fever and white blood cell count, followed by resolution of the initial imaging findings on follow-up imaging [18]. As stated above, antibiotic therapy should be initiated promptly after the cultures reveal the culprit microorganism, and of course tailored to that particular pathogen. Obtaining cultures directly from the abscess cavity is especially important when the abscess is of pyogenic origin [31]. To maximize the success of therapy, patients should remain on antibiotic therapy for at least 2 weeks, with the mean duration typically between 2 and 6 weeks. Most commonly, antibiotics should have activity against Gram-negative bacilli, Gram-positive cocci, and possibly anaerobes for maximal impact, but again, the selection of the proper antibiotic regimen should solely be based on the responsible pathogen [18].

For these small uniloculated abscesses, percutaneous drainage should only be considered if systemic antibiotic therapy fails. Note that it is imperative that a drain be inserted, as simple aspiration of the abscess cavity is unlikely to provide any benefit other than identifying the responsible pathogen. When a percutaneous drainage procedure was needed for patients who developed a liver abscess after TACE, the recovery time was relatively quick, at around 18 days until complete cure [4].

On the other hand, if the abscess is between 3 and 5 cm in diameter, percutaneous drainage is the recommended initial treatment. If such an approach is unsuccessful, surgical drainage becomes necessary to fully treat the abscess. Patients presenting with multi-loculated abscesses, regardless of their sizes, should be treated with surgical resection, as a less invasive approach is unlikely to successfully treat the patient.

## 6. Discussion

Our review confirms that locoregional therapies for patients with liver cancer do not frequently cause liver abscess under general conditions. However, for patients who had undergone any kind of prior hepatobiliary reconstructive surgery or biliary interventions that compromised the Ampulla of Vater, the risk of developing a liver abscess after a locoregional treatment increased significantly.

What is certain is that a history of hepatobiliary intervention increased the risk of developing a liver abscess as a complication of locoregional therapy. Here, preservation of the sphincter of Oddi is of the utmost importance in protecting patients from developing a liver abscess by preventing intestinal bacteria from entering the normally sterile biliary tree [30]. Any violation of the Ampulla of Vater through compromise of the sphincter of Oddi can lead to the colonization of the intrahepatic biliary tree by intestinal bacteria.

Procedures that either bypass or damage the sphincter of Oddi can create a pathway for bacterial entrance into the biliary tree, increasing the risk of liver abscess. Additionally, liver abscesses are more likely to occur in patients with bilioenteric anastomosis or biliary endoprosthesis, as these procedures can induce chronic colonization of the biliary tree [30]. All of the locoregional procedures used to treat patients with liver cancer, such as TACE, yttrium-90 radioembolization, radiofrequency ablation, microwave ablation, and laser-induced thermotherapy, destroy tumors by causing tissue necrosis as their main mechanism of action to destroy tumor tissue. The necrosis of the tumor and adjacent normal liver parenchyma provides a portal of entry for secondary infection [2]. Surgical or interventional radiology procedures that involve the biliary tree typically introduce bacteria into the normally sterile biliary ductal system. These procedures also compromise the Ampulla of Vater by damaging the sphincter of Oddi, increasing the risk of ascending infection (cholangitis and intrahepatic abscess) from the bowel into the intrahepatic biliary system. When patients with such risk factors are treated locoregionally, either percutaneously using thermal ablation or intraarterially in the form of TACE or Y90 radioembolotherapy, thereby causing tumor necrosis, the risk of developing an intrahepatic abscess is significantly increased precisely at the necrotic tumor sites.

Given the severity of a liver abscess, it is therefore critical to minimize if not prevent its occurrence. Indeed, mortality rates of intrahepatic abscesses ranged from 13.3% to 50% [4]. Clinical data to date are reassuring, as they show unequivocally that the combination of bowel preparation and prophylactic antibiotics did prevent the formation of liver abscess in most patients who were at high risk. For proper prophylactic antibiotics to be effective, the abscess types and bacterial profiles causing such infections must be accounted for. Pyogenic and amoebic abscesses are the two most common and relevant types that may spread to the liver from the small bowel through the biliary tree. Cholangitis occurs when bacteria ascend from the upper intestinal tract into the hepatobiliary tree due to a compromised Ampulla of Vater [32]. In such scenarios, antibiotic drug therapy must cover a broad spectrum of bacteria, including Gram-positive, Gram-negative, and Entamoeba histolytica species [32].

Multiple studies have confirmed the effectiveness of such prophylactic regimens in significantly minimizing if not preventing the formation of a liver abscess [32]. The addition of bowel preparation helps cleanse the bowel, relieve bowel pressure pre-procedure, and keep the flow of bacteria and other microbes downstream rather than possibly refluxing into the biliary tree.

Despite the success of prophylactic antibiotics for patients at high risk of developing a liver abscess, making a recommendation on the exact type of antibiotic drug regimen to be used remains difficult and somewhat elusive. This is largely due to the fact that dispensing antibiotics in such situations is generally under the control of the infectious disease department. As a result, when patients are considered for locoregional therapy but are at high risk for developing a liver abscess, it is imperative to prescribe an appropriate antibiotic regimen under the direction of the infectious disease department to ensure appropriate coverage and a successful outcome.

In the event that a patient does develop a liver abscess, it must be managed and treated aggressively with antibiotics and percutaneous drainage when appropriate. Microbial cultures can be used to identify infectious organisms to guide and ultimately select the appropriate antibiotic regimen.

## 7. Conclusions

Liver abscess is an uncommon complication of locoregional therapy for patients with liver cancer regardless of the type of therapy. However, patients who have undergone prior hepatobiliary manipulation are at a dramatically increased risk of developing a liver abscess after locoregional therapy, causing significant morbidity. Therefore, given such a risk, it is critically important to prophylactically treat patients with a combination of antibiotics and a bowel preparation to minimize or possibly eliminate the risk of an abscess. Existing data confirm the value of such a prophylactic approach.

## Figures and Tables

**Table 1:** Liver abscess incidence in patients without prior hepatobiliary intervention.

Treatment Modality	Incidence of Liver Abscess Across Studies
Chemoembolization	0.2–8.8%
Yttrium-90 Radioembolization	6.6%
Radiofrequency Ablation	0.3–1.8%
Microwave Ablation	0.4–1.6%
Laser-Induced Thermotherapy	0.7%
Radiation Therapy	3.6%
Surgical Resection	3.0%

**Table 2:** Liver abscess incidence in patients with prior hepatobiliary intervention.

Treatment Modality	Incidence of Liver Abscess Across Studies
Chemoembolization	12.1–85.7%
Radioembolization	7.1%
Radiofrequency Ablation (RFA)	22–100%
Microwave Ablation (MWA)	27.2–80%
Surgical Resection	10.6%
Yttrium-90 Radioembolization	0%

## Data Availability

No new data were created or analyzed in this study. Data sharing is not applicable to this article.
